# Short-term outcomes of mini-thoracotomy, multiportal video-assisted thoracic surgery, and multiportal robot-assisted thoracic surgery for complex segmentectomy

**DOI:** 10.1007/s11701-026-04088-9

**Published:** 2026-10-23

**Authors:** Tetsuya Isaka, Gentaro Murata, Naoki Nishina, Makoto Ogata, Ikki Takada, Chiaki Kanno, Takuya Nagashima, Hiroyuki Ito

**Affiliations:** Department of Thoracic Surgery, Kanagawa Cancer Center, 2-3-2 Nakao, Asahi, Yokohama, 241-8515 Kanagawa Japan

**Keywords:** Complex segmentectomy, Mini-thoracotomy, Robot-assisted thoracic surgery, Video-assisted thoracic surgery, Postoperative pain

## Abstract

This study examined the association of surgical approach with operative and postoperative outcomes as well as margin and nodal outcomes after complex segmentectomy, comparing mini-thoracotomy (MT) with multiportal minimally invasive thoracic surgery (mMITS) and, within mMITS, video-assisted thoracic surgery (mVATS) with multiportal robot-assisted thoracic surgery (mRATS), using propensity score matching (PSM). The study cohort comprised 808 consecutive patients treated with complex segmentectomy for suspected malignant pulmonary tumors from January 2015 through July 2026. Of these, 138 underwent MT and 670 mMITS, including 605 mVATS and 65 mRATS. PSM (1:2) was performed for MT versus mMITS (n = 126 vs. 252) and mVATS versus mRATS (n = 126 vs. 63). We assessed operative outcomes, postoperative complications, pain-related outcomes only for the mVATS–mRATS comparison, and margin and nodal outcomes. Blood loss was lower with mMITS than with MT (5 vs. 20 mL, p < 0.001), and chest tube duration was shorter (p = 0.002). Margin and nodal outcomes did not differ significantly between MT and mMITS. mRATS was associated with less blood loss than mVATS (3 vs. 5 mL, p = 0.006), a shorter duration of regular oral analgesic use (20 vs. 31 days, p = 0.007), and greater hilar and mediastinal lymph node yields (hilar, p = 0.037; mediastinal, p = 0.025). For complex segmentectomy, mMITS was associated with less blood loss and shorter chest tube duration than MT, without significant differences in margin and nodal outcomes. Within mMITS, mRATS was associated with shorter regular oral analgesic use and greater hilar and mediastinal lymph node yields than mVATS.

**Supplementary Information:**

The online version contains supplementary material available at https://doi.org/10.1007/s11701-026-04088-9.

## Introduction

Segmentectomy is a parenchyma-sparing surgical option for patients with early-stage primary lung cancer, pulmonary metastases, and indeterminate pulmonary nodules [1–3]. Compared with lobectomy, segmentectomy is associated with better postoperative pulmonary function preservation [1, 4], smaller postoperative changes in pulmonary artery pressure [5], and less deterioration in body composition [6]. Accordingly, segmentectomy is now accepted for selected patients with early-stage lung cancer [1, 7], with particular relevance to older patients and those with limited pulmonary function or significant comorbidities [8]. Consequently, the utilization of segmentectomy has recently increased [9].

The technical complexity varies substantially with the pattern of segmentectomy. Simple segmentectomy requires only one intersegmental plane, whereas complex segmentectomy requires multiple, more complex planes [10]. Compared with simple segmentectomy, complex segmentectomy encompasses more diverse resection patterns and requires more meticulous hilar dissection, peripheral vessel and bronchial handling, and intersegmental division, necessitating careful preoperative planning to secure an adequate margin in relation to tumor size [11–14]. Because of these technical demands, procedure-specific factors, including surgical exposure, instrument maneuverability, and stapler handling, may affect perioperative outcomes. Given these differences in technical complexity between simple and complex segmentectomy, combining the two procedures in a single analysis may obscure differences attributable to the surgical approach itself. Therefore, we focused specifically on complex segmentectomy to evaluate the influence of surgical approach in a technically demanding setting.

Mini-thoracotomy (MT) and minimally invasive thoracic surgery (MITS), including video-assisted thoracic surgery (VATS) and robot-assisted thoracic surgery (RATS), have become widely adopted in thoracic surgery [15]. Robotic systems offer enhanced instrument articulation and three-dimensional visualization, potentially facilitating technically complex thoracic procedures [16]. However, during segmentectomy, particularly complex segmentectomy, limited tactile feedback during MITS may make intraoperative tumor localization and intersegmental division more challenging than in open approaches [17]. Most previous studies comparing thoracic surgical approaches have focused on lobectomy, demonstrating the perioperative advantages of MITS over thoracotomy and the differences between RATS and VATS [18–24]. In contrast, evidence regarding differences among MT, VATS, and RATS for complex segmentectomy remains limited. To our knowledge, no previous study has compared MT, VATS, and RATS specifically for complex segmentectomy.

Therefore, this study aimed to compare MT and MITS (VATS and RATS) in patients undergoing complex segmentectomy using propensity score matching (PSM). We evaluated operative and postoperative outcomes as well as margin and nodal outcomes. Furthermore, VATS and RATS were compared within the MITS cohort to examine differences associated with the surgical approach.

## Materials and methods

### Patients

**Fig. 1:**
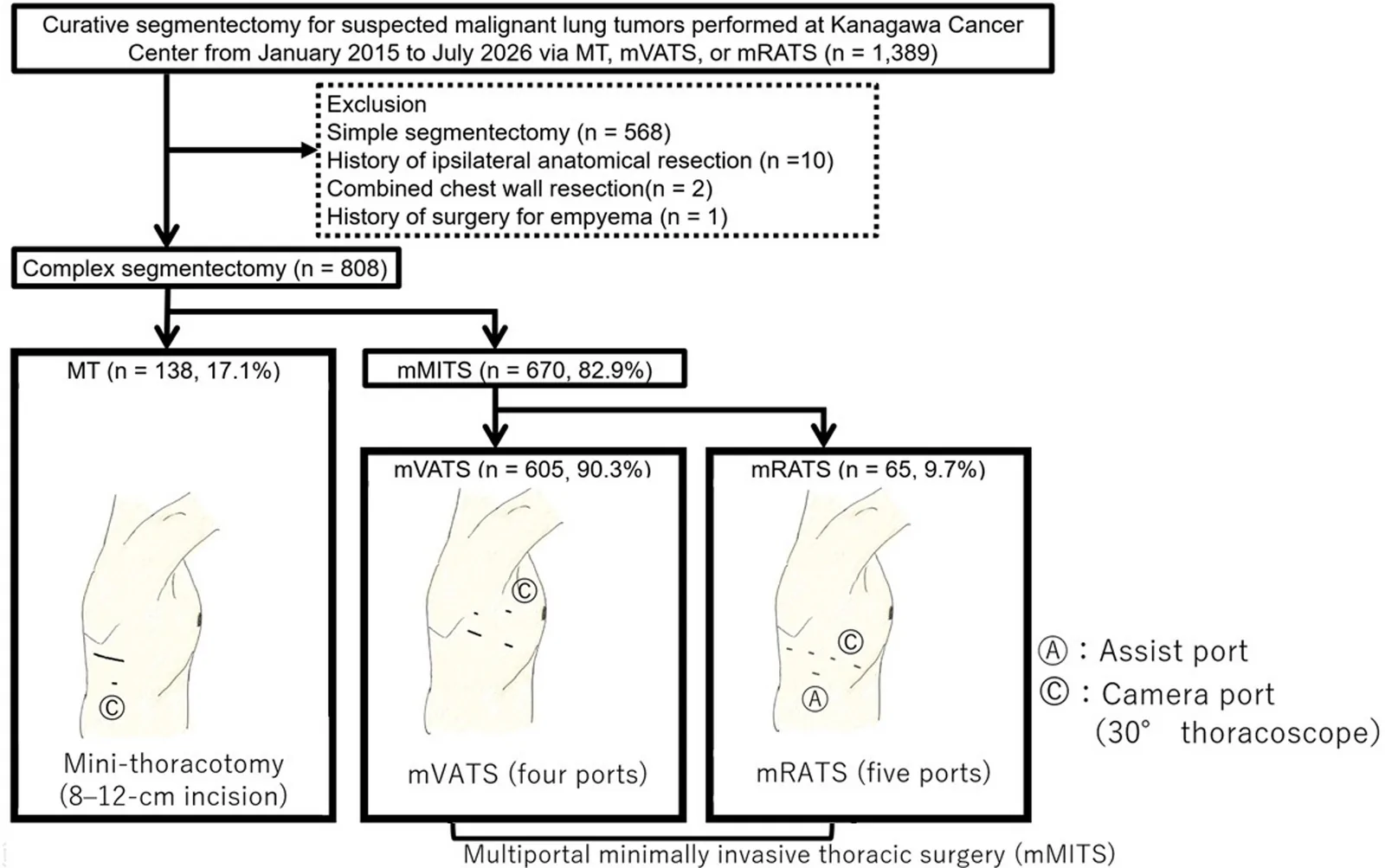
Patient selection and surgical approaches. Between January 2015 and July 2026, 1,389 patients underwent curative-intent segmentectomy for suspected malignant lung tumors. After predefined exclusions, 808 patients who underwent complex segmentectomy were included: 138 MT, 605 mVATS, and 65 mRATS. The lower panel shows the port placement and incision configuration for each surgical approach. A, assistant port; C, camera port

This retrospective, single-center observational cohort study included consecutive patients who underwent curative-intent segmentectomy for suspected malignant pulmonary lesions at Kanagawa Cancer Center between January 2015 and July 2026. Patients were identified from our institutional surgical database. During the study period, 1,389 patients underwent segmentectomy. After excluding patients who underwent simple segmentectomy (*n* = 568), had a history of previous anatomical lung resection (*n* = 10), underwent combined chest wall resection (*n* = 2), or had previous surgery for empyema (*n* = 1), 808 consecutive patients who underwent complex segmentectomy were included in the analysis (Fig. 1). The Institutional Review Board of the Kanagawa Cancer Center approved the study protocol on January 16, 2023 (No. 2022 Eki-118). Given the retrospective nature of the study, individual informed consent was waived, and an opt-out approach was used.

A segmentectomy was classified as complex unless it was an upper-division resection, lingulectomy, superior segmentectomy (S6), or basal segmentectomy completed with a single intersegmental plane (Supplementary Fig. 1) [10]. This study also included cases of subsegmentectomy and complex segmentectomy combined with adjacent subsegmental, segmental, or lobar resections.

For primary or suspected primary lung cancer, intentional segmentectomy was used for clinical N0 tumors no larger than 2 cm or for ground-glass opacity-predominant tumors no larger than 3 cm on computed tomography (CT). Compromised segmentectomy was chosen for patients with clinical N0 solid-dominant tumors who were considered unsuitable for lobectomy owing to advanced age, impaired cardiopulmonary function, or significant comorbidities. For metastatic or suspected metastatic lung tumors, segmentectomy was indicated for lesions for which adequate surgical margins were deemed difficult to achieve by wedge resection. In all cases, preoperative three-dimensional CT simulation using Synapse Vincent (Fujifilm, Tokyo, Japan) was performed, and segmentectomy was selected only when sufficient surgical margins were considered to be attainable.

In primary or suspected primary lung cancer showing a solid-dominant component, mediastinal nodes were generally assessed by systematic or selective dissection. Mediastinal lymph node sampling was permitted based on intraoperative judgment for compromised segmentectomy cases. Mediastinal lymph node dissection or sampling could be omitted for ground-glass opacity-predominant lesions.

### Surgical procedures and postoperative management

The port placement for MT, multiportal VATS (mVATS), and multiportal RATS (mRATS) is illustrated in Fig. 1. MT was performed using an 8–12-cm utility thoracotomy with an additional camera port. mVATS was performed using a four-port approach with a confronting upside-down monitor arrangement designed to reproduce the operative orientation of a thoracotomy [25]. mRATS was performed using four robotic ports placed within a single intercostal space, together with a separate assistant port. mVATS and mRATS were collectively defined as mMITS. All procedures were performed or supervised by board-certified thoracic surgeons, including certified thoracoscopic and robotic surgeons.

The intersegmental plane was identified primarily using intravenous indocyanine green (ICG) fluorescence with near-infrared imaging. After division of the target pulmonary arterial branches, 10 mg of ICG was administered intravenously, and the intersegmental demarcation line was identified and marked under near-infrared fluorescence imaging. In all cases, the intersegmental plane was divided using staplers.

Within mMITS, the same postoperative analgesic protocol was used for mVATS and mRATS. mMITS was managed with continuous intravenous fentanyl infusion and an intercostal nerve block, with fentanyl infusion terminated on postoperative day (POD) 1. Epidural analgesia was not used in patients undergoing mMITS. Postoperative pain was recorded daily using the Numeric Rating Scale (NRS) during routine clinical practice. For each patient, the highest NRS score recorded during PODs 0–1 and that recorded during PODs 2–6 were used for the respective analyses. During the immediate postoperative period before the resumption of oral intake, intravenous analgesics were administered. After oral intake was resumed, regular oral analgesics were prescribed. As-needed (PRN) analgesics were administered during hospitalization, and PRN use frequency was calculated as the mean number of administrations per hospitalization day.

Following discharge, postoperative pain was assessed during outpatient follow-up based on wound-related pain and analgesic requirements. Regular oral analgesic use was calculated over the interval between surgery and the end of the final prescription period, with the latter determined from the prescription date plus the prescribed number of days. Continuation or discontinuation of regular oral analgesics was determined during routine follow-up visits through discussions between the physician and the patient based on the patient’s postoperative pain status.

### Definitions and oncologic assessment

Complex segmentectomies were categorized as subsegmentectomy, single-segment complex segmentectomy, or combined/extended complex segmentectomy. Combined/extended complex segmentectomy included complex segmentectomy combined with additional subsegmental, segmental, or lobar resection. Lymph node dissection was categorized into four levels: none, hilar, selective mediastinal, and systematic mediastinal dissection. Hilar lymph node dissection accompanied by mediastinal nodal sampling was categorized as selective mediastinal lymph node dissection.

Complications after segmentectomy included events of Clavien–Dindo grade II or higher [26]. An air leak persisting for at least 5 days after surgery was considered prolonged. Intraoperative injury referred to inadvertent injury to the pulmonary artery, pulmonary vein, or bronchus sustained during the procedure.

Pathological tumor margin was defined as the minimum distance from the tumor edge to the resection margin, as measured on formalin-fixed specimens serially sectioned at 5-mm intervals. Tumor size was defined as the maximum axial tumor diameter on high-resolution CT with 1–2-mm slices in the lung window setting. The margin-to-tumor ratio (MTR) was calculated by dividing the pathological tumor margin by the tumor size.

For patients with primary non-small cell lung cancer (NSCLC), oncologic outcomes were additionally evaluated. Local recurrence was defined as recurrence at the resection margin or within the same lobe in which the segmentectomy was performed. Locoregional recurrence included local recurrence and recurrence in the ipsilateral hilar or mediastinal lymph nodes or ipsilateral chest wall. Distant recurrence included pleural dissemination and recurrence at other distant sites.

### Statistical analyses

Continuous and categorical variables were analyzed using the Mann–Whitney U test and Fisher’s exact test, respectively. To address selection bias and confounding, separate PSM analyses were conducted for MT versus mMITS and mVATS versus mRATS. A multivariable logistic regression model generated the propensity score from baseline variables comprising age, sex, smoking history, chronic lung disease history, tumor type, tumor size, consolidation tumor ratio (CTR), tumor laterality, resected segment location, preoperative percentage predicted vital capacity (%VC), and preoperative FEV1%. Patients were matched 1:2 using nearest-neighbor matching without replacement. The caliper was set at 0.2 standard deviations of the logit-transformed propensity score. Standardized mean differences (SMDs) were used to evaluate covariate balance after matching.

Pain-related outcomes were compared only between mVATS and mRATS because the same postoperative analgesic protocol was used for both approaches, and were not compared between MT and mMITS because their analgesic protocols differed. All baseline variables included in the PSM analyses were complete. For postoperative outcomes, available data were analyzed without imputation when values were missing. Statistical significance was defined by *p* < 0.05. All statistical analyses were performed by T.I. using EZR on R Commander, version 1.30 (Saitama Medical Center, Jichi Medical University, Saitama, Japan).

## Results

Overall, 808 patients underwent complex segmentectomy, including 138 who underwent MT and 670 who underwent mMITS, comprising 605 and 65 mVATS and mRATS patients, respectively. The most frequently performed complex segmentectomies were left S1 + 2 (*n* = 159), right S2 (*n* = 119), and right S3 (*n* = 96); the detailed distribution of individual segmentectomies according to surgical approach is provided in Supplementary Table 1. Intraoperative injuries occurred in one MT patient (0.7%), 11 mVATS patients (1.8%) and one mRATS patient (1.5%). These included pulmonary artery injury/bleeding in six patients (one MT and five mVATS), bronchial injury or inadvertent bronchial division in six patients (five mVATS and one mRATS), and inadvertent pulmonary vein division in one mVATS patient. Among the five pulmonary artery injuries in the mVATS group, two required conversion to thoracotomy for hemostasis, whereas the remaining three were managed without conversion.

Before PSM, the MT and mMITS groups differed in CTR, resected segment location, and preoperative %VC (CTR, *p* < 0.001; resected segment location, *p* < 0.001; %VC, *p* = 0.030). No differences were observed in the other baseline clinical characteristics (Table 1). The mMITS group exhibited lower blood loss and earlier chest tube removal than the MT group, and the distribution of complex segmentectomy types differed significantly between the groups (*p* = 0.007) (Table 2).

**Table 1:** Patient characteristics before and after propensity score matching: MT versus mMITS

Variable	Before PSM (unmatched)			After PSM (matched)			
	MT **(n = 138)**	mMITS **(n = 670)**	*p* value ^a)^	MT **(n = 126)**	mMITS **(n = 252)**	*p* value ^a)^	SMD
Age (years), median (IQR)	72 (64–76)	72 (64–77)	0.526 ^b)^	72 (65–76)	71 (61–76)	0.228 ^b)^	0.081
Male, n (%)	66 (47.8)	339 (50.6)	0.576	59 (46.8)	130 (51.6)	0.445	0.095
Smoking history (ever), n (%)	77 (55.8)	394 (58.8)	0.570	70 (55.6)	150 (59.5)	0.507	0.080
Chronic lung disease history, n (%)	23 (16.7)	141 (21.0)	0.295	22 (17.5)	44 (17.5)	1.000	< 0.001
Type of the tumor, n (%)							
Primary lung cancer	105 (76.1)	540 (80.6)		97 (77.0)	193 (76.6)		
Metastatic lung cancer	28 (20.3)	96 (14.3)		24 (19.0)	45 (17.9)		
Benign	5 (3.6)	34 (5.1)	0.191	5 (4.0)	14 (5.6)	0.819	0.078
Tumor size (mm), median (IQR)	17 (13–23)	17 (13–23)	0.662 ^b)^	17 (13–23)	17 (13–24)	0.639 ^b)^	0.036
Consolidation tumor ratio (%), median (IQR)	20 (0–56)	55 (23–100)	< 0.001 ^b)^	22 (0–60)	25 (9–50)	0.723 ^b)^	0.022
Right side, n (%)	85 (61.6)	384 (57.3)	0.394	78 (61.9)	156 (61.9)	1.000	< 0.001
Location, n (%)							
Upper lobe	67 (48.6)	491 (73.3)		67 (53.2)	138 (54.8)		
Lower lobe	71 (51.4)	179 (26.7)	< 0.001	59 (46.8)	114 (45.2)	0.827	0.032
Preoperative %VC (%), median (IQR)	107 (95–117)	110 (99–120)	0.030 ^b)^	107 (96–117)	107 (97–118)	0.824 ^b)^	0.034
Preoperative FEV1% (%), median (IQR)	76 (70–81)	74 (68–79)	0.110 ^b)^	76 (69–80)	74 (68–80)	0.294 ^b)^	0.032

(a) Fisher’s exact test; (b) Mann–Whitney U test

**FEV1%**, FEV1/FVC ratio; **IQR**, interquartile range; **mMITS**, multiportal minimalཌཙ invasive thoracic surgery; **MT**, mini-thoracotomy; **PSM**, propensity score matching; **SMD**, standardized mean difference; **%VC**, percentage predicted vital capacity

**Table 2:** Operative, postoperative, margin, and nodal outcomes before and after propensity score matching: MT versus mMITS

Variable	Before PSM (unmatched)			After PSM (matched)		
	MT **(n = 138)**	mMITS **(n = 670)**	*p* value ^a)^	MT **(n = 126)**	mMITS **(n = 252)**	*p* value ^a)^
**Operative outcomes**						
Operation time (min), median (IQR)	161 (136–195)	157 (132–187)	0.112 ^b)^	161 (136–197)	160 (133–196)	0.414 ^b)^
Blood loss (ml), median (IQR)	20 (5–40)	5 (3–10)	< 0.001 ^b)^	20 (5–39)	5 (3–10)	< 0.001 ^b)^
Intraoperative injury, n (%)	1 (0.7)	12 (1.8)	0.708	1 (0.8)	4 (1.6)	0.669
Conversion to thoracotomy, n (%)	—	6 (0.9)		—	6 (2.4)	
Types of complex segmentectomy, n (%)						
Subsegmentectomy	5 (3.6)	64 (9.6)		5 (4.0)	15 (6.0)	
Single-segment complex segmentectomy	76 (55.1)	405 (60.4)		68 (54.0)	149 (59.1)	
Combined/extended complex segmentectomy ^c)^	57 (41.3)	201 (30.0)	0.007	53 (42.1)	88 (34.9)	0.348
Extent of lymph node dissection, n (%)						
No lymph node dissection	28 (20.3)	163 (24.3)		22 (17.5)	62 (24.6)	
Hilar lymph node dissection	88 (63.8)	408 (60.9)		84 (66.7)	148 (58.7)	
Selective mediastinal lymph node dissection	17 (12.3)	93 (13.9)		15 (11.9)	40 (15.9)	
Systematic mediastinal lymph node dissection	5 (3.6)	6 (0.9)	0.083	5 (4.0)	2 (0.8)	0.044
**Postoperative outcomes**						
Postoperative complications ^d)^, n (%)						
Air leak grade ≥ IIIa	2 (1.4)	39 (5.8)	0.032	2 (1.6)	19 (7.5)	0.017
Prolonged air leak (≥ 5 days)	5 (3.6)	27 (4.0)	1.000	5 (4.0)	13 (5.2)	0.799
Pneumonia grade ≥ II	2 (1.4)	12 (1.8)	1.000	2 (1.6)	6 (2.4)	0.724
Reoperation	1 (0.7)	3 (0.4)	0.528	1 (0.8)	3 (1.2)	1.000
Death within 90 days	0	3 (0.4)	1.000	0	2 (0.8)	0.554
Chest tube removal (postoperative day), median (IQR)	2 (2–3)	2 (2–2)	< 0.001 ^b)^	2 (2–3)	2 (2–2)	0.002 ^b)^
Chest tube removal (≤ POD 2), n (%)	82 (59.4)	515 (77.0)	< 0.001	75 (59.5)	194 (77.0)	0.001
**Margin and nodal outcomes**						
Pathological tumor margin (mm), median (IQR)	11 (6–15)	12 (8–19)	0.070 ^b)^	11 (6–17)	11 (6–15)	0.751 ^b)^
Pathological margin positive, n (%)	6 (4.3)	9 (1.3)	0.293	5 (4.0)	6 (2.4)	0.517
Margin-to-tumor ratio, median (IQR)	0.7 (0.4–1.1)	0.7 (0.4–1.2)	0.242 ^b)^	0.6 (0.4–1.0)	0.7 (0.4–1.1)	0.583 ^b)^
Number of hilar lymph nodes dissected, median (IQR)	0 (0–2)	0 (0–3)	0.079 ^b)^	0 (0–2)	0 (0–3)	0.227 ^b)^
Number of mediastinal lymph nodes dissected, median (IQR)	0 (0–0)	0 (0–0)	0.268 ^b)^	0 (0–0)	0 (0–0)	0.520 ^b)^

(a) Fisher’s exact test; (b) Mann–Whitney U test; (c) Combined/extended complex segmentectomy included complex segmentectomy with additional subsegmental resection, resection involving two or more complex segments, or concomitant simple segmentectomy or lobectomy; (d) Clavien–Dindo classification

**IQR**, interquartile range; **mMITS**, minimally invasive thoracic surgery; **MT**, mini-thoracotomy; **POD**, postoperative day; **PSM**, propensity score matching

The matched cohort consisted of 378 patients (MT, *n* = 126; mMITS, *n* = 252). After matching, adequate balance was obtained across all baseline covariates, with absolute SMDs < 0.1 (Table 1). The distribution of complex segmentectomy types did not differ significantly between the MT and mMITS groups after matching (*p* = 0.348) (Table 2). Conversion to thoracotomy occurred in six patients (2.4%) in the mMITS group. Operative time was similar between MT and mMITS (161 vs. 160 min, *p* = 0.414), while blood loss was lower with mMITS (20 vs. 5 mL, *p* < 0.001). Grade ≥ IIIa air leak was more frequent after mMITS (1.6% vs. 7.5%, *p* = 0.017). However, chest tube duration was shorter with mMITS (*p* = 0.002), and chest tube removal by POD 2 was more frequent (59.5% vs. 77.0%, *p* = 0.001). No group differences were found in prolonged air leak, pneumonia, reoperation, or 90-day mortality (Table 2).

Pathological tumor margin (11 [IQR, 6–17] vs. 11 [IQR, 6–15] mm, *p* = 0.751), margin positivity (4.0% vs. 2.4%, *p* = 0.517), and MTR (0.6 [IQR, 0.4–1.0] vs. 0.7 [IQR, 0.4–1.1], *p* = 0.583) did not differ significantly between the MT and mMITS groups. Hilar and mediastinal lymph node yields also did not differ significantly between the groups (Table 2).

In the comparison between mVATS and mRATS before PSM, the two groups were generally similar in baseline characteristics, although right-sided lesions were more frequent in the mRATS group (56.0% vs. 69.2%, *p* = 0.048) (Table 3). Intraoperative injuries occurred in 11 mVATS patients (1.8%) and one mRATS patient (1.5%). Six patients in the mVATS group required conversion to thoracotomy, including two due to intraoperative bleeding, whereas no conversions occurred in the mRATS group. The mRATS group demonstrated lower blood loss, a shorter duration of regular oral analgesic use, and higher hilar and mediastinal lymph node yields than the mVATS group, whereas the distribution of procedure types and other perioperative and pain-related outcomes did not differ significantly between the groups (Table 4).

**Table 3:** Clinicopathological characteristics before and after propensity score matching: mVATS versus mRATS

Variable	Before PSM (unmatched)			After PSM (matched)			
	mVATS (*n* = 605)	mRATS (*n* = 65)	*p* value	mVATS (*n* = 126)	mRATS (*n* = 63)	*p* value	SMD
Age (years), median (IQR)	72 (64–77)	72 (65–77)	0.995 ^b)^	71 (64–78)	72 (65–77)	0.950 ^b)^	< 0.001
Male, n (%)	309 (51.1)	30 (46.2)	0.514	63 (50.0)	30 (47.6)	0.877	0.048
Smoking history (ever), n (%)	361 (59.7)	33 (50.8)	0.185	68 (54.0)	33 (52.4)	0.878	0.032
Chronic lung disease history, n (%)	122 (20.2)	19 (29.2)	0.108	33 (26.2)	17 (27.0)	1.000	0.018
Type of the tumor, n (%)							
Primary lung cancer	493 (81.5)	47 (72.3)		96 (76.2)	47 (74.6)		
Metastatic lung cancer	84 (13.9)	12 (18.5)		22 (17.5)	11 (17.5)		
Benign	28 (4.6)	6 (9.2)	0.146	8 (6.3)	5 (7.9)	0.891	0.062
Tumor size (mm), median (IQR)	17 (13–23)	16 (13–20)	0.423 ^b)^	15 (11–20)	16 (13–21)	0.303 ^b)^	0.095
Consolidation tumor ratio (%), median (IQR)	55 (22–100)	55 (26–100)	0.360 ^b)^	59 (23–100)	55 (26–100)	0.909 ^b)^	< 0.001
Right side, n (%)	339 (56.0)	45 (69.2)	0.048	89 (70.6)	43 (68.3)	0.740	0.052
Location, n (%)							
Upper lobe	447 (73.9)	44 (67.7)		88 (69.8)	43 (68.3)		
Lower lobe	158 (26.1)	21 (32.3)	0.302	38 (30.2)	20 (31.7)	0.868	0.034
Preoperative %VC (%), median (IQR)	110 (99–120)	108 (95–118)	0.127 ^b)^	107 (94–118)	108 (96–118)	0.849 ^b)^	0.014
Preoperative FEV1% (%), median (IQR)	74 (68–79)	75 (69–80)	0.941 ^b)^	74 (69–79)	74 (69–80)	0.936 ^b)^	0.003

(a) Fisher’s exact test; (b) Mann–Whitney U test

**FEV1%**, FEV1/FVC ratio; **IQR**, interquartile range; **mRATS**, multiportal robot-assisted thoracoscopic surgery; **mVATS**, multiportal video-assisted thoracoscopic surgery; **PSM**, propensity score matching; **SMD**, standardized mean difference; **%VC**, percentage predicted vital capacity

**Table 4:** Operative and postoperative outcomes, and margin and nodal outcomes, before and after propensity score matching: mVATS versus mRATS

Variable	Before PSM (unmatched)			After PSM (matched)		
	mVATS **(n = 605)**	mRATS **(n = 65)**	*p* value ^a)^	mVATS **(n = 126)**	mRATS **(n = 63)**	*p* value ^a)^
**Operative outcomes**						
Operation time (min), median (IQR)	157 (131–188)	155 (136–175)	0.654 ^b)^	161 (135–200)	157 (136–177)	0.308 ^b)^
Blood loss (ml), median (IQR)	5 (3–10)	3 (3–5)	0.010 ^b)^	5 (3–10)	3 (3–5)	0.006 ^b)^
Intraoperative injury, n (%)	11 (1.8)	1 (1.5)	1.000	4 (3.2)	1 (1.6)	0.666
Conversion to thoracotomy, n (%)	6 (1.0)	0	1.000	4 (3.2)	0	0.303
Types of complex segmentectomy, n (%)						
Subsegmentectomy	58 (9.6)	6 (9.2)		12 (9.5)	5 (7.9)	
Single-segment complex segmentectomy	373 (61.7)	32 (49.2)		77 (61.1)	31 (49.2)	
Combined/extended complex segmentectomy ^c)^	174 (28.8)	27 (41.5)	0.091	37 (29.4)	27 (42.9)	0.191
Extent of lymph node dissection, n (%)						
No lymph node dissection	149 (24.6)	14 (21.5)		35 (27.8)	14 (22.2)	
Hilar lymph node dissection	372 (61.5)	36 (55.4)		69 (54.8)	34 (54.0)	
Selective mediastinal lymph node dissection	79 (13.1)	14 (21.5)		20 (15.9)	14 (22.2)	
Systematic mediastinal lymph node dissection	5 (0.8)	1 (1.5)	0.203	2 (1.6)	1 (1.6)	0.683
**Postoperative outcomes**						
Postoperative complications ^d)^, n (%)						
Air leak grade ≥ IIIa	35 (5.8)	4 (6.2)	0.784	11 (8.7)	4 (6.3)	0.777
Prolonged air leak (≥ 5 days)	26 (4.3)	1 (1.5)	0.503	10 (7.9)	1 (1.6)	0.103
Pneumonia grade ≥ II	11 (1.8)	1 (1.5)	1.000	4 (3.2)	1 (1.6)	0.666
Reoperation	3 (0.5)	0	1.000	0	0	1.000
All (grade ≥ II)	86 (14.2)	7 (10.8)	0.572	20 (15.9)	7 (11.1)	0.509
Death within 90 days	3 (0.5)	0	1.000	0	0	1.000
Chest tube removal (postoperative day), median (IQR)	2 (2–2)	2 (2–3)	0.953 ^b)^	2 (2–3)	2 (2–3)	0.662 ^b)^
Chest tube removal (≤ POD 2), n (%)	467 (77.2)	48 (73.8)	0.538	92 (73.0)	47 (74.6)	0.863
Maximum NRS PODs 0–1, median (IQR)	5 (5–7)	5 (4–7)	0.463^b)^	6 (5–7)	5 (4–7)	0.249^b)^
Maximum NRS PODs 2–6, median (IQR)	2 (0–3)	2 (0–3)	0.888^b)^	2 (0–3)	2 (0–3)	0.605^b)^
As-needed (PRN) analgesic use during hospitalization (times/day), median (IQR)	0.3 (0.1–0.6)	0.3 (0.1–0.5)	0.540 ^b)^	0.3 (0.1–0.7)	0.3 (0.1–0.5)	0.236 ^b)^
Duration of regular oral analgesic use (days), median (IQR)	30 (19–44)	22 (15–40)	0.010 ^b)^	31 (20–47)	20 (15–38)	0.007 ^b)^
**Margin and nodal outcomes**						
Pathological tumor margin (mm), median (IQR)	12 (7–19)	13 (9–18)	0.590 ^b)^	10 (6–16)	13 (9–18)	0.103 ^b)^
Pathological margin positive, n (%)	9 (1.5)	0	1.000	2 (1.6)	0	0.553
Margin-to-tumor ratio, median (IQR)	0.7 (0.4–1.2)	0.8 (0.5–1.2)	0.320 ^b)^	0.7 (0.4–1.1)	0.8 (0.5–1.2)	0.393 ^b)^
Number of hilar lymph nodes dissected, median (IQR)	0 (0–2)	1 (0–3)	0.002 ^b)^	0 (0–3)	2 (0–4)	0.037 ^b)^
Number of mediastinal lymph nodes dissected, median (IQR)	0 (0–0)	0 (0–2)	0.001 ^b)^	0 (0–0)	0 (0–2)	0.025 ^b)^

(a) Fisher’s exact test; (b) Mann–Whitney U test; (c) Combined/extended complex segmentectomy included complex segmentectomy with additional subsegmental resection, resection involving two or more complex segments, or concomitant simple segmentectomy or lobectomy; (d) Clavien–Dindo classification

**IQR**, interquartile range; **mRATS**, multi-portal robot-assisted thoracoscopic surgery; **mVATS**, multi-portal video-assisted thoracoscopic surgery; **NRS**, Numeric Rating Scale; **POD**, postoperative day; **PRN**, pro re nata (as needed); **PSM**, propensity score matching

Following PSM, the analysis included 189 patients, comprising 126 and 63 patients in the mVATS and mRATS groups, respectively. After matching, adequate balance was obtained across all baseline covariates, with absolute SMDs < 0.1 (Table 3). The distribution of complex segmentectomy types did not differ significantly between the groups after matching (*p* = 0.191). Conversion to thoracotomy was observed in four mVATS patients (3.2%) but in no mRATS patients (*p* = 0.303) (Table 4). Operative time did not differ significantly between the groups (161 vs. 157 min, *p* = 0.308); however, blood loss was lower in the mRATS group (5 vs. 3 mL, *p* = 0.006). Regular oral analgesic use was of shorter duration in the mRATS group (31 vs. 20 days, *p* = 0.007). Postoperative complications, NRS scores during PODs 0–6, PRN analgesic use, and chest tube duration did not differ between the groups. Pathological tumor margin (10 [IQR, 6–16] vs. 13 [IQR, 9–18] mm, *p* = 0.103), margin positivity (1.6% vs. 0%, *p* = 0.553), and MTR (0.7 [IQR, 0.4–1.1] vs. 0.8 [IQR, 0.5–1.2], *p* = 0.393) did not differ significantly between the mVATS and mRATS groups. Hilar and mediastinal lymph node yields were significantly greater in the mRATS group than in the mVATS group (hilar, *p* = 0.037; mediastinal, *p* = 0.025) (Table 4).

Among the 638 patients with primary NSCLC, 522 (81.8%) had pathological stage 0–IA2 disease, and 16 (2.5%) had pathological nodal involvement. Overall, 555 patients (87.0%) were pN0 without lymphatic or vascular invasion. During a median follow-up of 27.0 months (IQR, 11.3–49.0), local recurrence occurred in 5 patients (0.8%), locoregional recurrence in 19 (3.0%), and distant recurrence in 16 (2.5%). Detailed oncologic characteristics, margin-related outcomes, and recurrence patterns according to surgical approach are summarized in Supplementary Table 2.

## Discussion

The comparison between MT and mMITS showed less blood loss and earlier chest tube removal with mMITS, while operative time and postoperative complications were generally comparable, and no significant differences were observed in margin and nodal outcomes. Within the mMITS cohort, mRATS and mVATS had comparable operative time and postoperative complications, with no significant differences in margin-related outcomes; however, mRATS was associated with lower blood loss, a shorter duration of regular oral analgesic use, and higher hilar and mediastinal lymph node yields. These findings provide comparative data on three major surgical approaches for complex segmentectomy.

Previous studies on complex segmentectomy performed using the MT, VATS, and RATS approaches are summarized in Table 5 [3, 10, 12–14, 27–30]. Although acceptable perioperative outcomes have generally been reported, the available evidence remains limited and heterogeneous with respect to surgical access, including uniportal, multiportal, hybrid VATS, and mini-thoracotomy approaches. In addition, reported findings regarding perioperative outcomes and the potential advantages of RATS over VATS have not been entirely consistent, and direct comparisons across MT, VATS, and RATS for complex segmentectomy remain scarce. The present study adds to this limited evidence by evaluating 808 consecutive complex segmentectomies and performing stepwise propensity score-matched comparisons of MT versus mMITS and mVATS versus mRATS.

**Table 5:** Previous studies on complex segmentectomy performed using each approach

Approaches	Previous studies (year) [Reference]	*N*	Access	Operative time (min)	Blood loss (mL)	Chest tube removal (days)	Conversion to thoracotomy	Overall complications^a)^	PAL^d)^	Pneumonia	Tumor margin distance (cm)
RATS	Okazaki (2024) [27]	48	Multiportal	169	10		0.0%	2.1%	0.0%^e)^	2.1%	2.5
	Lee (2024) [3]	245	N.R.	177			0.8%	25.7%^b)^	5.3%^f)^	14.7%	
	Xie (2019) [28]	66	Multiportal	131	53	2.7			6.1%^g)^	3.0%	
	Nakamura (2026) [29]	162	Multiportal	230	10	2	0.0%	9.9%	4.3%^f)^	1.2%	
	Present study	65	Multiportal	155	3	2	0.0%	10.8%	1.5%	1.5%	1.3
VATS	Chen (2021) [30]	179	Uni/multiportal	180	66	2.9	0.9%	6.1%^b)^	5.0%^f)^	2.2%	
	Ahn (2023) [14]	132	Uniportal	105	50	1	0.0%	3.0%^b)^	0.8%^f)^	0.8%	2.0
	Bédat (2019) [12]	111	Multiportal	145		1	3.9%	30.0%^c)^		7.0%	
	Xie (2019) [28]	61	Biportal	133	49	2.8	0.0%		8.2%^g)^	3.3%	
	Nakamura (2026) [29]	106	Multiportal	186	20	2	0.0%	20.8%	2.8%^f)^	4.7%	
	Present study	605	Multiportal	157	5	2	1.0%	14.2%	4.3%	1.8%	1.2
MT or hybrid VATS	Handa (2020) [10]	99	Hybrid	178	50	2		26.3%	11.1%^h)^		1.7
	Okubo (2021) [13]	251	MT	113	20			2.0%^b)^	0.8%^g)^	0.4%	2.2
	Present study	138	MT	161	20	2		21.7%	3.6%	1.4%	1.1

a) Clavien–Dindo Grade ≥ II

b) Grading not specified

c) Clavien–Dindo Grade ≥ I

d) Prolonged air leak (≥ 5 days)

e) Prolonged air leak (≥ 7 days)

f) Definition of PAL not specified

g) Prolonged air leak (≥ 6 days)

h) Prolonged air leak (≥ 8 days)

**MT**, mini-thoracotomy; **N.R**., not reported; **PAL**, Prolonged air leak; **RATS**, robot-assisted thoracoscopic surgery; **VATS**, video-assisted thoracoscopic surgery

Previous studies comparing thoracotomy and MITS, primarily conducted in the setting of lobectomy, have demonstrated reduced postoperative invasiveness with MITS, including lower rates of prolonged air leak, shorter drainage duration, shorter hospitalization, less postoperative pain, and reduced blood loss, while maintaining comparable postoperative morbidity and survival outcomes [18, 19, 21]. However, Agzarian et al. reported a longer operative time with RATS than with thoracotomy [18]. In addition, Kneuertz et al. reported less frequent lymph node upstaging following VATS than after thoracotomy, raising concerns about the adequacy of nodal assessment with VATS [20]. Nevertheless, whether these findings are applicable to complex segmentectomy remains unclear.

Owing to the advantages of direct visualization and tactile feedback, MT has conventionally been selected for technically demanding cases. However, the present findings suggest that, even for technically demanding complex segmentectomy, MITS may offer certain perioperative advantages over MT without significant differences in most postoperative complications. Although intraoperative blood loss was lower with mMITS, the absolute difference was modest. The shorter chest tube duration observed with mMITS may represent a potential perioperative advantage. No significant differences were observed in pathological tumor margin, margin positivity, or MTR between MT and mMITS. However, these findings should be interpreted cautiously, and the long-term oncologic implications of the observed margin-related outcomes require further evaluation.

Several prospective studies have compared RATS and VATS within MITS. The ROMAN trial (*n* = 77) and the RVlob trial (*n* = 320) [22, 23], which mainly focused on lobectomy, showed similar perioperative outcomes between the two approaches, whereas RATS was associated with a greater lymph node yield. By contrast, the J-RATSIG-01 study (*n* = 405), wherein lobectomy accounted for approximately 72% and 83% of the procedures in the RATS and VATS groups, respectively, reported a longer operative time with RATS and suggested a potential advantage of VATS regarding postoperative pain [24]. However, as these studies largely evaluated lobectomy, whether their findings can be directly applied to complex segmentectomy remains uncertain.

For complex segmentectomy, previous studies comparing RATS with VATS have differed in surgical access and reported perioperative outcomes. Xie et al. compared 66 mRATS and 61 biportal VATS cases without PSM and found no significant differences in operative time or postoperative complications; lymph node yield was also similar within the atypical segmentectomy subgroup [28]. Nakamura et al. analyzed 268 complex segmentectomies (mVATS, *n* = 106; mRATS, *n* = 162) and, after PSM, compared 99 matched pairs. mRATS was associated with a longer operative time but fewer Clavien–Dindo grade ≥ 2 complications (10.1% vs. 20.2%, *p* = 0.047) and postoperative arrhythmias (1.0% vs. 7.1%, *p* = 0.030) [29]. Tang et al. compared 271 uniportal VATS and 91 mRATS cases and, after PSM, analyzed 81 matched pairs. mRATS was associated with a longer operative time (133 vs. 92 min, *p* < 0.001), lower major morbidity (0% vs. 7.4%, *p* = 0.031), and greater lymph node yield [31]. Taken together, these previous studies suggest that the relative advantages of mRATS and VATS in complex segmentectomy remain uncertain.

In this study, postoperative complications were comparable between mVATS and mRATS, and no significant differences were observed in pathological margin-related outcomes. Although mRATS was associated with statistically lower intraoperative blood loss, the absolute difference was small and unlikely to be clinically meaningful. Notably, the same postoperative analgesic protocol was used for mVATS and mRATS. Within this context, mRATS was associated with a shorter duration of regular oral analgesic use than mVATS. This difference may partly reflect differences in intercostal access and instrument mechanics between the two approaches. In our procedures, mRATS generally involved two intercostal spaces for port placement, whereas mVATS involved two to four intercostal spaces. In addition, the remote center of the robotic instruments is positioned at the chest wall, which may reduce mechanical stress on the intercostal spaces during instrument manipulation. These features may reduce postoperative chest wall pain; however, the underlying mechanism cannot be determined from the present study.

The mRATS group also had greater hilar and mediastinal lymph node yields than the mVATS group. This finding may reflect technical characteristics of robotic assistance, including improved instrument articulation and stable three-dimensional visualization. As hilar lymph node dissection during segmentectomy is technically demanding [32], these features may facilitate hilar and mediastinal lymph node dissection during complex segmentectomy. However, this finding should be interpreted cautiously because of the retrospective study design and the possibility of residual confounding.

The novelty of the present study lies in its stepwise comparison of MT, mVATS, and mRATS for complex segmentectomy using PSM within a large consecutive cohort. To our knowledge, no previous study has directly compared these three approaches specifically in this setting. In addition, we evaluated pain-related and multiple margin-related outcomes, including pathological tumor margin, margin positivity, and MTR, which have been insufficiently addressed in previous studies. However, this study has several limitations. First, its retrospective, single-center design leaves the possibility of selection bias and residual confounding despite PSM. Differences in treatment era, surgeon experience, and learning-curve effects may also have influenced the outcomes. In an exploratory early-versus-late analysis, no consistent learning-curve–related improvement was observed across the three approaches, except that prolonged air leak decreased in the late phase of mVATS while operative time increased (Supplementary Table 3). The absence of a clear learning-curve effect may partly reflect that the procedures were performed or supervised by experienced thoracic surgeons with expertise in thoracoscopic and robotic surgery. To further assess historical bias, we performed a sensitivity analysis restricted to the period when mVATS and mRATS were performed concurrently. The main findings of shorter regular oral analgesic use and greater hilar and mediastinal lymph node yields with mRATS were also observed in this sensitivity analysis (Supplementary Table 4), suggesting that these differences were not explained solely by treatment era. However, because the three approaches were not performed under identical conditions with respect to treatment era and surgeon experience, residual confounding related to these factors cannot be excluded. Second, the small mRATS cohort may have limited the statistical power and precision of the comparisons between mRATS and mVATS. Third, because technical complexity may vary among individual resection patterns within complex segmentectomy, further studies are needed to determine the most appropriate surgical approach for specific resection patterns. Finally, this study focused primarily on short-term perioperative outcomes as well as margin and nodal outcomes; therefore, long-term recurrence and survival according to surgical approach require further evaluation.

## Conclusion

In patients undergoing complex segmentectomy for suspected malignant lung tumors, mMITS was associated with less blood loss and shorter chest tube duration than MT, without significant differences in margin and nodal outcomes. Within the mMITS cohort, mRATS was associated with a shorter duration of regular oral analgesic use and greater hilar and mediastinal lymph node yields than mVATS.

## Supplementary Information

Below is the link to the electronic supplementary material.

Supplementary Material 1

Supplementary Material 2

## Data Availability

The datasets generated and/or analyzed during the current study are available from the corresponding author on reasonable request.

## Declarations

### Supplementary Fig. 1. Definition of complex segmentectomy

Complex segmentectomy included segmentectomies other than superior segmentectomy (S6), basal segmentectomy, lingulectomy, and upper-division segmentectomy. Representative examples include right S3, right S10, left S1 + 2, and left S8.
