# Supplementary material for: Short-term outcomes of mini-thoracotomy, multiportal video-assisted thoracic surgery, and multiportal robot-assisted thoracic surgery for complex segmentectomy
Source: J Robot Surg. 2026 Oct 23;20(1):1128. doi: 10.1007/s11701-026-04088-9 (PMC13694486; doi:10.1007/s11701-026-04088-9)
Supplement: Supplementary file 1 [file 11701_2026_4088_MOESM2_ESM.docx]

**Supplementary Tables**

Supplementary Table 1. Distribution of resected segments according to surgical approach before propensity score matching

| **Lobe** | **Resected segment** ^a)^ | **MT**  **(n = 138)** | **mVATS**  **(n = 605)** | **mRATS**  **(n = 65)** | **Total**  **(n = 808)** |
| --- | --- | --- | --- | --- | --- |
| Right upper lobe | S1 | 10 | 64 | 5 | 79 |
|  | S2 | 16 | 88 | 15 | 119 |
|  | S3 | 15 | 73 | 8 | 96 |
|  | S1+2 | 1 | 17 | 3 | 21 |
| Right lower lobe | S6 (subsegmentectomy) | 0 | 5 | 1 | 6 |
|  | S7 | 4 | 4 | 0 | 8 |
|  | S7+S8 | 0 | 3 | 0 | 3 |
|  | S7+S8+S9 | 4 | 0 | 0 | 4 |
|  | S8 | 18 | 34 | 5 | 57 |
|  | S8+9 | 5 | 14 | 3 | 22 |
|  | S9+S10 | 1 | 15 | 2 | 18 |
|  | S10 | 11 | 22 | 3 | 36 |
| Left upper lobe | S1+2 | 13 | 136 | 10 | 159 |
|  | S3 | 11 | 64 | 3 | 78 |
|  | S4 | 1 | 3 | 0 | 4 |
|  | S5 | 0 | 2 | 0 | 2 |
| Left lower lobe | S6  (subsegmentectomy) | 0 | 4 | 0 | 4 |
|  | S8 | 8 | 16 | 2 | 26 |
|  | S8+9 | 4 | 6 | 1 | 11 |
|  | S9 | 0 | 4 | 0 | 4 |
|  | S9+10 | 3 | 7 | 3 | 13 |
|  | S10 | 13 | 24 | 1 | 38 |

a) Subsegmentectomies and segmentectomies combined with adjacent subsegmental resection were classified according to the corresponding segment. For example, S1 with additional S2a subsegmentectomy was classified as S1, and S3b subsegmentectomy was classified as S3. When a complex segmentectomy was combined with a simple segmentectomy, classification was based on the complex segmentectomy component.

**mRATS**, multiportal robot-assisted thoracic surgery; **MT**, mini-thoracotomy; **mVATS**, multiportal video-assisted thoracic surgery; **S**, pulmonary segment.

Supplementary Table 2. Oncologic characteristics and outcomes of patients with primary NSCLC

| Variable | Overall NSCLC (n=638) | MT (n=104) | mVATS (n=487) | mRATS (n=47) |
| --- | --- | --- | --- | --- |
| Pathological tumor characteristics |  |  |  |  |
| Tumor size (mm), median (IQR) | 17 (13–23) | 16 (14–22) | 17 (13–24) | 18 (13–21) |
| Consolidation tumor ratio (%), median (IQR) | 53 (22–90) | 21 (0–57) | 64 (28–100) | 55 (29–96) |
| Primary tumor SUVmax, median (IQR) ^a)^ | 1.8 (1.0–3.9) | 1.1 (0–2.6) | 1.9 (1.1–4.0) | 2.0 (1.2–4.0) |
| Histology, n (%) |  |  |  |  |
| Adenocarcinoma | 575 (90.1) | 89 (85.6) | 445 (91.4) | 41 (87.2) |
| Non-adenocarcinoma | 63 (9.9) | 15 (14.4) | 42 (8.6) | 6 (12.8) |
| Pathological stage, n (%) |  |  |  |  |
| 0–IA2 | 522 (81.8) | 84 (80.8) | 396 (81.3) | 42 (89.4) |
| IA3–IB | 91 (14.3) | 14 (13.5) | 73 (15.0) | 4 (8.5) |
| ≥II | 25 (3.9) | 6 (5.8) | 18 (3.7) | 1 (2.1) |
| Pathological N0, n (%) | 622 (97.5) | 100 (96.2) | 476 (97.7) | 46 (97.9) |
| Lymphatic invasion (Ly+), n (%) | 26 (4.1) | 5 (4.8) | 19 (3.9) | 2 (4.3) |
| Vascular invasion (V+), n (%) | 69 (10.8) | 14 (13.5) | 51 (10.5) | 4 (8.5) |
| pN0 with Ly0/V0, n (%) | 555 (87.0) | 86 (82.7) | 427 (87.7) | 42 (89.4) |
| Margin and nodal outcomes |  |  |  |  |
| Pathological tumor margin (mm), median (IQR) | 13 (8–20) | 12 (9–19) | 13 (8–20) | 13 (9–18) |
| Margin positive, n (%) | 13 (2.0) | 6 (5.8) | 7 (1.4) | 0 (0.0) |
| Margin-to-tumor ratio, median (IQR) | 0.7 (0.4–1.2) | 0.7 (0.4–1.1) | 0.7 (0.4–1.2) | 0.7 (0.4–1.1) |
| Margin-to-tumor ratio <1, n (%) | 406 (63.6) | 61 (58.7) | 314 (64.5) | 31 (66.0) |
| Extent of lymph node dissection, n (%) |  |  |  |  |
| No lymph node dissection | 120 (18.8) | 11 (10.6) | 102 (20.9) | 7 (14.9) |
| Hilar lymph node dissection | 408 (63.9) | 74 (71.2) | 305 (62.6) | 29 (61.7) |
| Selective mediastinal lymph node dissection | 99 (15.5) | 14 (13.5) | 75 (15.4) | 10 (21.3) |
| Systematic mediastinal lymph node dissection | 11 (1.7) | 5 (4.8) | 5 (1.0) | 1 (2.1) |
| Hilar lymph nodes dissected, median (IQR) | 3 (1–5) | 5 (3–8) | 3 (1–5) | 4 (2–6) |
| Mediastinal lymph nodes dissected, median (IQR) | 0 (0–0) | 0 (0–0.75) | 0 (0–0) | 0 (0–2) |
| Recurrence outcomes |  |  |  |  |
| Local recurrence, n (%) | 5 (0.8) | 1 (1.0) | 4 (0.8) | 0 (0.0) |
| Locoregional recurrence, n (%) | 19 (3.0) | 6 (5.8) | 13 (2.7) | 0 (0.0) |
| Distant recurrence, n (%) | 16 (2.5) | 6 (5.8) | 10 (2.1) | 0 (0.0) |

a) Primary tumor SUVmax was analyzed using available data (overall, n=530; MT, n=91; mVATS, n=400; mRATS, n=39).

**IQR**, interquartile range; **mRATS**, multiportal robot-assisted thoracic surgery; **MT**, mini-thoracotomy; **mVATS,** multiportal video-assisted thoracic surgery; **NSCLC**, non-small cell lung cancer.

Supplementary Table 3. Exploratory comparison of perioperative and technical outcomes between the early and late phases according to surgical approach

| **Variable** | **MT early (n=69)** | **MT late (n=69)** | **p value** | **mVATS early (n=302)** | **mVATS late (n=303)** | **p value** | **mRATS early (n=32)** | **mRATS late (n=33)** | **p value ^a)^** |
| --- | --- | --- | --- | --- | --- | --- | --- | --- | --- |
| Operative outcomes |  |  |  |  |  |  |  |  |  |
| Operation time (min), median (IQR) | 168  (139–205) | 159  (136–183) | 0.118 ^b)^ | 151  (129–174) | 168  (134–200) | <0.001 ^b)^ | 166  (139–178) | 149  (129–172) | 0.067 ^b)^ |
| Blood loss (mL), median (IQR) | 20 (10–40) | 15 (5–35) | 0.427 ^b)^ | 5 (3–10) | 5 (3–5) | 0.293 ^b)^ | 3 (3–5) | 3 (3–5) | 0.588 ^b)^ |
| Intraoperative injury, n (%) | 0 (0.0) | 1 (1.4) | 1.000 | 7 (2.3) | 4 (1.3) | 0.383 | 1 (3.1) | 0 (0.0) | 0.492 |
| Conversion to thoracotomy, n (%) | 0 (0.0) | 0 (0.0) | 1.000 | 4 (1.3) | 2 (0.7) | 0.450 | 0 (0.0) | 0 (0.0) | 1.000 |
| Postoperative outcomes |  |  |  |  |  |  |  |  |  |
| Air leak grade ≥ IIIa ^c)^, n (%) | 0 (0.0) | 2 (2.9) | 0.496 | 19 (6.3) | 16 (5.3) | 0.607 | 3 (9.4) | 1 (3.0) | 0.355 |
| Prolonged air leak (≥5 days), n (%) | 3 (4.3) | 2 (2.9) | 1.000 | 18 (6.0) | 8 (2.6) | 0.047 | 0 (0.0) | 1 (3.0) | 1.000 |
| All postoperative complications grade ≥ II ^c)^, n (%) | 11 (15.9) | 19 (27.5) | 0.148 | 49 (16.2) | 37 (12.2) | 0.164 | 6 (18.8) | 1 (3.0) | 0.054 |
| Chest tube duration (days), median (IQR) | 2 (2–3) | 2 (2–3) | 0.913 ^b)^ | 2 (2–2) | 2 (2–2) | 0.856 ^b)^ | 2 (2–2) | 2 (2–3) | 0.311 ^b)^ |

a) Fisher’s exact test; b) Mann–Whitney U test; c) Clavien–Dindo classification

**mRATS**, multiportal robot-assisted thoracoscopic surgery; **MT**, mini-thoracotomy; **mVATS**, multiportal video-assisted thoracoscopic surgery.

Supplementary Table 4. Sensitivity analysis for potential historical bias comparing mVATS and mRATS during the period in which both approaches were performed concurrently

| **Variable** | **mVATS (n=473)** | **mRATS (n=65)** | **p value** ^a)^ |
| --- | --- | --- | --- |
| Operative outcomes |  |  |  |
| Operation time (min), median (IQR) | 159 (132–190) | 155 (136–175) | 0.476 ^b)^ |
| Blood loss (mL), median (IQR) | 3 (3–5) | 3 (3–5) | 0.143 ^b)^ |
| Intraoperative injury, n (%) | 7 (1.5) | 1 (1.5) | 1.000 |
| Conversion to thoracotomy, n (%) | 2 (0.4) | 0 (0.0) | 1.000 |
| Types of complex segmentectomy, n (%) |  |  | 0.189 |
| Subsegmentectomy | 47 (9.9) | 6 (9.2) |  |
| Single-segment complex segmentectomy | 282 (59.6) | 32 (49.2) |  |
| Combined/extended complex segmentectomy | 144 (30.4) | 27 (41.5) |  |
| Postoperative outcomes |  |  |  |
| Air leak grade ≥ IIIa, n (%) | 27 (5.7) | 4 (6.2) | 0.780 |
| Prolonged air leak (≥5 days), n (%) | 18 (3.8) | 1 (1.5) | 0.716 |
| All postoperative complications grade ≥ II ^c)^, n (%) | 59 (12.5) | 7 (10.8) | 0.841 |
| Chest tube duration (days), median (IQR) | 2 (2–2) | 2 (2–3) | 0.866 ^b)^ |
| Duration of regular oral analgesic use (days), median (IQR) | 29 (19–43) | 22 (15–40) | 0.017 ^b)^ |
| Margin and nodal outcomes |  |  |  |
| Pathological tumor margin (mm), median (IQR) | 13 (8–20) | 13 (9–18) | 0.973 ^b)^ |
| Margin-to-tumor ratio, median (IQR) | 0.8 (0.4–1.2) | 0.8 (0.5–1.2) | 0.656 ^b)^ |
| Number of hilar lymph nodes dissected, median (IQR) | 0 (0–3) | 1 (0–3) | 0.036 ^b)^ |
| Number of mediastinal lymph nodes dissected, median (IQR) | 0 (0–0) | 0 (0–2) | 0.005 ^b)^ |

a) Fisher’s exact test; b) Mann–Whitney U test; c) Clavien–Dindo classification

**IQR**, interquartile range; **mRATS**, multiportal robot-assisted thoracoscopic surgery; **mVATS**, multiportal video-assisted thoracoscopic surgery.
